# Changes in food and nutrient intakes in Korean adults before and during the COVID-19 pandemic: data from the 2011-2020 Korea National Health and Nutrition Examination Survey

**DOI:** 10.4178/epih.e2023015

**Published:** 2023-02-01

**Authors:** Kyungwon Oh, Suyeon Park, Sihyun Park, Sungha Yun, Hongseok Choi, Eun Kyeong Jeong

**Affiliations:** 1 Division of Health and Nutrition Survey and Analysis, Bureau of Chronic Disease Prevention and Control, Korea Disease Control and Prevention Agency, Cheongju, Korea; 2 Bureau of Chronic Disease Prevention and Control, Korea Disease Control and Prevention Agency, Cheongju, Korea; 3 Public Health Care Headquarters, Seoul National University Bundang Hospital, Seongnam, Korea

**Keywords:** COVID-19 pandemic, Korea National Health and Nutrition Exam ination Survey, Dietary habits, Food, Nutrients

## Abstract

**OBJECTIVES:**

This study was to examine the changes in dietary habits and food and nutrient intakes between before (2019) and during (2020) the coronavirus disease 2019 (COVID-19) pandemic from the Korea National Health and Nutrition Examination Survey (KNHANES).

**METHODS:**

A total of 54,995 participants aged ≥19 years who participated in the 2011-2020 KNHANES were included. The 10-year trend (2011-2020) and differences between 2019 and 2020 for dietary habits and food and nutrient intakes were estimated using SAS.

**RESULTS:**

In the past 10 years (2011-2020), the dietary habits (increase in skipping meals and eating out), food intake (increase in meats and decrease in fruits and vegetables), and nutrient intake (increase in fat and decrease in sodium) in adults have changed. When comparing between 2019 and 2020, there were 4.6%p decrease in the eating out more than once a day. On the other hand, there were no significant differences in the intakes of food, energy and most of nutrients between 2019 and 2020, except for the proportion of energy intake from fat (1.0%p increase) and carbohydrate (1.0%p decrease).

**CONCLUSIONS:**

Although a change in dietary habits from before to during the COVID-19 pandemic was observed, food and nutrient intakes have not deteriorated markedly and appear similar to the trends in the past 10 years. Throughout the COVID-19 pandemic, it is necessary to monitor the effects of changes in dietary habits on health as well as food and nutrient intakes.

## INTRODUCTION

Social distancing from the coronavirus disease 2019 (COVID19) pandemic in 2020 has impacted the food choices, food purchasing and preparation, dietary habits, quantities and qualities of diet, as well as the food and nutrient intakes, of households and individuals [1-3]. Nutritional imbalance decreases the functioning of the immune system and increases the risks of COVID-19 itself, progression to severe COVID-19, and chronic diseases, such as obesity, diabetes, and hypertension [4,5]. Because chronic diseases are also risk factors for COVID-19 infection and severe symptoms thereof [6], international organizations such as the World Health Organization and governments around the world are recommending healthy eating habits, such as drinking plenty of water and increases in intakes of fruits and vegetables, and protein of healthy sources to prevent COVID-19 transmission, given the importance of nutrition in preventing and managing chronic and infectious diseases [7].

Since the COVID-19 outbreak, studies on changes in eating habits and nutritional status have been extensively conducted using various methods and on various populations, but contrasting results have been published. While some studies reported that dietary habits have deteriorated since the COVID-19 pandemic began, as demonstrated by the increased frequencies of meals and snacks, decreased intakes of fruits and vegetables, and increased intakes of processed foods and sugars [8-13], other studies [14,15] have reported improved dietary habits, such as increased fruit and vegetable intakes and improved diet quality, as an attempt to prevent and recover from COVID-19. Favorable and unfavorable changes in dietary habits that varied depending on individual characteristics, including age, income level, and health behaviors were also reported even within a study [8,16,17]. To date, most of studies have been conducted using a simple online questionnaire with a biased sample, and cohort studies using a validated nutritional survey are limited by a small sample size [3].

In Korea, changes in dietary and physical activity behaviors from the COVID-19 pandemic have been reported in adolescents, such as an increase in skipping meals, a decrease in eating out, and a decrease in fruit intake [18]. Even in adults, especially men, physical activity decreased and the prevalence of chronic diseases, such as obesity and hypercholesterolemia, increased markedly during the COVID-19 pandemic [19,20]. In terms of dietary habits, it has been reported that 38.5% and 21.5% of the subjects increased food delivery and processed food consumption, respectively, from before the COVID-19 pandemic [21], but results from studies analyzing the changes in food and nutrient intakes have not yet been published.

This study used data from the Korea National Health and Nutrition Examination Survey (KNHANES) in the last 10 years (2011- 2020) to examine the 10-year trends and changes between before (2019) and during (2020) the COVID-19 pandemic in dietary habits and food and nutrient intakes.

## MATERIALS AND METHODS

### Study population

The KNHANES is a national health survey that has been conducted annually by the Korea Disease Control and Prevention Agency (KDCA) since 1998 to estimate the health and nutritional status of Korean population. To represent the Korean population, a two-step stratified cluster sampling method is selected for about 200 primary sampling units (PSU) and 20-25 households per PSU each year [22]. Subjects were all over one-year-old and members of the household that was sampled using the method described above. For our analysis, we include a total of 54,995 people aged ≥ 19 years who completed the 2011-2020 KNHANES. From 2011-2019, the survey was completed for all PSU sampled, but in 2020, only 166 of 192 PSU (86%) were surveyed due to the COVID-19 pandemic. There were no significant differences in the gender, age, or income level of the participants between 2019 and 2020.

### Nutrition survey

The KNHANES consists of a health examination, a health interview, and a nutrition survey. The nutrition survey is divided into a dietary behavior survey, a 24-hour dietary recall, and a food security survey, and the team of dietitians visits the households of participants and conducts individual interviews with all the household members. The dietary behavior survey includes skipping meals, eating out, and eating together. For the 24-hour dietary recall, all food consumed the day before the survey is investigated in chronological order along with the location and type of meal, and name and amount of food consumed. To accurately determine the amount of consumption, each individual’s intake is investigated using measuring aids, such as a two-dimensional model book, measuring cup, and measuring spoon. Energy and nutrient intakes are calculated using the nutrient database (DB) for each food, which was established based on the National Standard Food Composition Table [23].

The proportion of skipping breakfast, eating out more than once a day, and eating with family at dinner were calculated from the dietary behavior survey and the proportion of households with food security was evaluated from the food security survey. The intakes for food, energy, and nutrient were calculated from the 24-hour dietary recall. This study focused on the intakes of grains, vegetables, fruits, meats, and beverages, as these demonstrated considerable differences in the amount of consumption across year. As for the nutrients, the focus was on the energy, fat, protein, carbohydrates, calcium, sodium, iron, vitamin A, riboflavin, and vitamin C intakes, which were the components for the objectives of the fifth National Health Plan (Health Plan 2030). As the unit of reference intake for vitamin A changed from μg RE to μg RAE in the Dietary Reference Intake for Koreans 2015, vitamin A intake was evaluated from 2016 in this study. To examine the changes in energy composition, the proportions of energy intake for the energy-source nutrients: fat, carbohydrates, and protein from 2011 to 2020 were presented. As meal type have been classified into homecooked meal, restaurant meal (eating at the restaurants; takeout or meal delivery), food services meal at school or workplace, and convenience food (ready-to-cook food, ready-to-eat food, etc.) since 2016, the proportions of energy intake from meal type were calculated from 2016 to 2020.

### Statistical analysis

The statistical analysis was performed using SAS, version 9.4 (SAS Institute Inc., Cary, NC, USA). All results were applied the sampling weights assigned to participants to represent the Korean population. Sampling weights were generated by considering complex sample design, non-response rate of the target population, and post-stratification. To adjust differences in results from changes in the age structure of each year, age-standardized results were calculated using the age-specific and sex-specific structures of the estimated population based on the 2005 population projections for Korea. The age-standardized mean value for dietary habits and food and nutrient intakes were calculated using PROC SURVEYREG and PROC SURVEYLOGISTIC. The analysis for 10-year changes and differences in estimates between before (2019) and during (2020) the COVID-19 pandemic were performed using PROC SURVEYREG.

### Ethics statement

This study was approved by the Institutional Review Board of the KDCA (2011-2014; 2018-2020). For the certain year (2015-2017) was waived by the Act (Article 2, Paragraph 1) and Enforcement Regulation (Article 2, Paragraph 2, item 1) of Bio-ethics and Safety Act.

## RESULTS

A total of 54,995 individuals, including 23,137 men and 31,858 women, completed the nutrition survey of the 2011-2020 KNHANES. The gender, age, and income level distributions are presented in Table 1.

According to the trend in dietary habits over the last 10 years (2011-2020), the proportion of skipping breakfast continued to increase, from 22.5% in 2011 to 36.7% in 2020 (Table 2). The proportion of eating out at least once per day tended to increase only in women, and the proportion of households with food security tended to increase, reaching 96.3% in 2020. When comparing between 2019 and 2020, the proportion of skipping breakfast in 2020 was 36.7%, which is an increase of 2.6%p from 2019 (34.1%), but this was not statistically significant. In 2020, the proportion of eating out at least once per day was 26.4%, a 4.6%p decrease (5.2%p in men, 3.8%p in women) from 2019 (31.0%), which was different from the 2011-2020 trend, and the proportion of households with food security demonstrated no difference between 2019 and 2020.

The intakes of grains, vegetables, and fruits decreased, whereas the intakes of meat and non-alcoholic beverages increased from 2011-2020 (Table 3). In particular, the intake of fruits decreased by about 50 g, from 178 g in 2011 to 125 g in 2020, and that of non-alcoholic beverages doubled, from 130 g in 2011 to 254 g in 2020, a trend that was observed in both men and women. For examining the difference between 2019 and 2020, the intakes of grains, fruits, and vegetables decreased, and the intakes of beverages and meat increased between 2019 and 2020, which are similar to the trends from 2011-2020, but this difference was not statistically significant.

The energy intake decreased by about 200 kcal in men and 100 kcal in women in the last 10 years (Table 4). The proportion of energy intake from fat continued to increase, reaching 24.1% in 2020, and that from carbohydrate decreased to 60.1% in 2020. The energy intake in 2020 was 1,953 kcal, approximately a 40 kcal decrease (57 kcal in men, 20 kcal in women) from 2019, but this difference was not statistically significant. The proportion of energy intake from fat increased by 1.0%p, whereas that from carbohydrate decreased by 1.0%p in 2020 from 2019, and these demonstrated the same trends from 2011-2020. The proportions of energy intake from home-cooked meal (women) and food services meal (men) decreased, whereas that from restaurant meal (women) and convenience food (both men and women) increased from 2016 to 2020. The proportions of energy intake from homecooked meal, restaurant meal, food services meal, and convenience food in 2020 were 39.9%, 31.9%, 3.8%, and 9.7%, respectively. Unlike the trends from 2016 to 2020, the proportion of energy intake from home-cooked meal increased (2.5%p) in 2020 from 2019, and that from restaurant meal decreased, but this difference was not statistically significant. From 2019 to 2020, the proportion of energy intake from food services meal and convenience food decreased (2.0%p) and increased (1.2%p), respectively, which differences are consistent with the 2016-2020 trends.

From 2011-2020, the intakes of sodium, iron, and vitamin C have consistently decreased, whereas the intake of riboflavin has increased (Table 5). The vitamin C (women) and iron intakes (both men and women) decreased from 2019 to 2020, and the riboflavin intake (men) increased, maintaining the trend from 2011-2020. There were no differences in the calcium, sodium, and vitamin A intakes between 2019 and 2020 for both men and women.

## DISCUSSION

Analyzing the food and nutrient intakes in Korean adults from 2011-2020 KNHANES, dietary habits (increase in skipping meals, eating out), food intake (decreased intake of vegetables and fruits, increased intake of non-alcoholic beverages, and meat, etc.), and nutrient intake (increased fat intake, decrease in sodium, vitamin C, etc.) trends are similar to the past 20 years (1998-2020), as published in a previous study [23]. It is believed that this is due to gradual changes in the social environment related to diet, such as changes in household structure, development of food processing technology, and diversification of options for eating out. In other countries, there has also been a nutritional transition due to changes in income, urbanization, food technological advances, and nutritional policies due to economic and demographic changes [24]. In Japan, fat intake has continuously increased since the early 2000s, as did in Korea, reaching 28.6% in 2019, whereas the sodium intake has decreased [25,26]. Europe has observed a positive change over the past 30 years, with an increase in the intakes of fruits, vegetables, and fish and a decrease in the intakes of saturated fatty acids and sugars [27]. Even in the United States, macronutrient composition and dietary quality have generally improved including intakes of high-quality carbohydrates, plant protein, and polyunsaturated fatty acids since 2000, although there are still nutritional problems of high intakes of saturated fatty acids and low quality carbohydrates [28]. In contrast, with the exception of the improvement observed in the sodium intake since the KNHANES was first conducted in 1998, trends in most of dietary habits and nutrient intakes have worsened, suggesting that active nutritional interventions should be implemented.

Comparing the diets in adults before (2019) and during (2020) the COVID-19 pandemic, we observed changes in dietary habits, such as an increase in home-cooked meal and convenience food and a decrease in eating out (restaurant or food services). This seems to be due to social distancing, such as changes in work patterns and restrictions on social gatherings and multi-use facilities, and this is similar to the results of other domestic and overseas studies. According to the 2020 Consumer Behavior Survey for Food [29], the proportion of adults eating out decreased from 2019 by 7.5%p, and this trend continued in 2021 [30]. In overseas studies [10,31], the proportion of eating out decreased and the consumptions of home-cooked and processed foods increased due to COVID-19 pandemic ensued. Additionally, meal delivery increased during social distancing [10], and in the 2020 Korea Health Community Survey [21], the proportion reporting an increase in food delivery (38.5%) was higher than that reporting a decrease in food delivery (11.2%) in 2020 compared to 2019. In the light of this finding, the proportion of energy intake from restaurant meal by location where meal was consumed (restaurant or home) was compared in this study. The proportion of restaurant decreased by 3.3%p from 2019 to 2020 (23.6% in 2019; 20.3% in 2020), but the proportion of home through takeout or meal delivery increased by 2.1%p (6.5% in 2019; 8.6% in 2020; data not shown), which are results that are similar to those of previous studies [10,21].

Furthermore, various findings have been published on changes in dietary habits due to the COVID-19 pandemic. Many studies have shown undesirable changes, such as an increased frequency of meals and snacks [9,12] and late-night snacks [9], an increased intake of energy-dense foods [11], and a deterioration in overall eating habits and diet quality [13,32], resulting in weight gain [10,12]. In other studies [14,15], positive changes were reported, which included reducing sugar and fast-food consumption, in consideration of antioxidant consumption for infection prevention, anti-inflammatory, and immune function improvement. These inconsistent findings are attributable to differences in social distancing stage depending on the severity of the COVID-19 pandemic [13,33], area of residence [16], household characteristics, income, and individual health behaviors such as physical activity [17]. Additional in-depth research on the changes in dietary habits and food and nutrient intakes according to the characteristics of the COVID-19 pandemic in Korea, socioeconomic status, and health behaviors need to be conducted.

In this study, there were no significant differences in food, energy, and nutrient intakes before (2019) and during (2020) the COVID-19 pandemic. When the proportions of energy intakes by meal type were compared between 2019 and 2020, restaurant meal (-1.5%p) and food services meal (-2.0%p) decreased, whereas home-cooked meal (2.5%p) increased. The extent of these was small and may not have had a significant impact on the food and nutrient intakes in 2020 compared to 2019. In terms of the proportion by energy-source nutrient, the proportion of energy intake from fat increased from 2019 to 2020, whereas that from carbohydrate decreased. The fat intake in 2020 was 52.6 g, which is a 1.5 g increase from 2019 (51.1 g). However, this increase was not significant (p= 0.23), so it is probable that this difference in the proportion of energy from fat was due to a decrease in the energy intake (by approximately 40 kcal). In addition, the decrease in vitamin C intake (particularly in women) and the increase in riboflavin (particularly in men) in 2020 from 2019 seems to partially reflect the trend observed in the fruit intake (women: 18.3 g decrease, p= 0.09) and meat intake (men: 6.0 g increase, p= 0.42). Additionally, there were no significant differences in the proportion of population with adequate intakes of sodium, calcium, and vitamin A, and the proportion of population with excessive intake in energy and fat or deficient intake in energy, calcium, iron, vitamin A, and riboflavin between 2019 and 2020 (data not shown). The results of studies conducted overseas that used 24-hour dietary recall, dietary record, and food-frequency questionnaire were similar to those of this study. In the National Diet and Nutrition Survey [34] conducted in United Kingdom, energy intake slightly decreased, but there was no difference in food and nutrient intakes from 2019 to 2020. A study conducted in Spain on healthy postmenopausal women also demonstrated no difference in either energy or nutrient intake [35], and a study conducted in the United States with adults also reported an increase in energy density but no difference in energy intake [11]. In German children and adolescents, energy intake decreased by a negligible percentage (0.85%) from before the COVID-19 pandemic with a non-significant decrease in sugar sweetened beverages and ultra-processed foods intake [36]. Based on these results, it can be seen that efforts were made to maintain healthy eating habits to improve immune function to prevent COVID-19.

It is indeed encouraging to see that the trend in the food and nutrient intakes in Korea has generally remained unchanged despite the COVID-19 pandemic. Nevertheless, the results of this study is from the first year since the COVID-19 pandemic and, in the light of the ongoing pandemic, it is possible that the changes in dietary habits may independently, or in conjunction with other health behaviors, such as alcohol drinking or smoking, lead to significant changes in food and nutrient intakes to contribute to the increase in the prevalence of chronic diseases. It is suggestive that, whereas the physical activity level decreased in 2020, the first year of the COVID-19 pandemic, and slightly increased again in 2021, according to the Korea Youth Risk Behavior Survey, dietary habits such as skipping meals and the intakes of fruits and vegetables have continuously deteriorated in 2021 [18]. It is necessary to monitor whether dietary changes in the second year (2021) and third year (2022) of the COVID-19 pandemic will continue and whether these changes will affect food and nutrient intakes, and to produce the evidence for nutritional intervention to prevent the deterioration in trends.

The KNHANES has been conducted by dietitians who have completed training in the same methods (24-hour dietary recall) since the survey began in 1998. Since the main purpose of the KNHANES is to estimate the nutritional status of the current year, it uses the latest recipe DB and nutrient DB for each food to calculate the results. In other words, when calculating food and nutrient intakes for 2011-2012, 2013-2015, and 2016-2020, the 7th, 8th, and 9th editions of the latest National Standard Food Composition Table, respectively, were used. Although it is beneficial because the results reflect the latest nutritional information when the nutritional status is evaluated, there is the effect of the new DB needs to be considered when evaluating the food and nutrient intake trends.

## CONCLUSION

As a result of analyzing the food and nutrient intake trends in adults using the data from the KNHANES, there were changes in the food and nutrient intake patterns, such as increases in the intakes of meat and non-alcoholic beverages, and the proportion of energy intake from fat, and decreases in the intakes of grains, vegetables, fruits, vitamin C, and sodium for the past 10 years (2011- 2020). There were changes in dietary habits between before and during the COVID-19 pandemic, such as a decrease in eating out and an increase in the consumption for home-cooked meals, but food and nutrient intakes has largely remained unchanged without deterioration. Although it is encouraging that food and nutrient intakes has not deteriorated due to the COVID-19 pandemic, it is necessary to monitor the effects of changes in dietary habits on the food and nutrient intakes, during the COVID-19 pandemic.

## Figures and Tables

**Table 1.** General characteristics of the participants in this study, Korean men and women aged 19 years or older, using data from the 2011-2020 Korea National Health and Nutrition Examination Survey

Characteristics		2011	2012	2013	2014	2015	2016	2017	2018	2019	2020
Total		5,894 (100)	5,570 (100)	5,450 (100)	5,323 (100)	5,308 (100)	5,439 (100)	5,723 (100)	5,708 (100)	5,775 (100)	4,805 (100)
Gender											
	Men	2,434 (49.4)	2,231 (49.3)	2,269 (49.5)	2,199 (49.6)	2,249 (49.4)	2,245 (49.7)	2,484 (49.7)	2,443 (49.8)	2,498 (49.8)	2,085 (49.6)
	Women	3,460 (50.6)	3,339 (50.7)	3,181 (50.5)	3,124 (50.4)	3,059 (50.6)	3,194 (50.3)	3,239 (50.3)	3,265 (50.2)	3,277 (50.2)	2,720 (50.4)
Age (yr)											
	19-29	643 (19.0)	582 (18.6)	673 (18.3)	567 (18.3)	661 (18.4)	623 (17.7)	653 (17.5)	682 (17.6)	669 (17.3)	624 (17.3)
	30-39	1,054 (20.7)	937 (20.2)	964 (19.7)	908 (19.2)	718 (18.6)	966 (18.3)	845 (17.9)	862 (17.5)	855 (17.1)	633 (16.5)
	40-49	1,027 (21.8)	901 (21.6)	1,064 (21.3)	899 (21.0)	904 (20.6)	1,014 (20.8)	1,028 (20.4)	1,052 (19.9)	1,036 (19.4)	808 (19.0)
	50-59	1,145 (18.1)	1,034 (18.7)	1,002 (19.3)	995 (19.6)	1,081 (19.7)	939 (19.9)	1,117 (19.9)	1,055 (19.8)	1,061 (20.0)	861 (19.7)
	60-69	995 (10.6)	1,013 (10.7)	874 (10.9)	926 (11.3)	960 (11.8)	920 (12.4)	1,037 (12.9)	998 (13.5)	1,046 (14.1)	892 (14.9)
	≥70	1,030 (9.8)	1,103 (10.2)	873 (10.5)	1,028 (10.7)	984 (10.9)	977 (10.9)	1,043 (11.3)	1,059 (11.7)	1,108 (12.2)	987 (12.6)
Household income^1^											
	Low	1,157 (22.6)	1,087 (22.2)	1,068 (19.9)	1,074 (21.1)	1,052 (20.2)	1,091 (20.8)	1,150 (19.6)	1,146 (20.4)	1,151 (19.3)	963 (19.4)
	Low-middle	1,182 (20.6)	1,119 (21.3)	1,088 (19.9)	1,041 (19.8)	1,071 (20.3)	1,064 (18.9)	1,155 (20.4)	1,141 (20.8)	1,129 (19.6)	967 (19.3)
	Middle	1,167 (19.9)	1,078 (20.3)	1,108 (20.7)	1,054 (19.3)	1,038 (19.6)	1,077 (20.0)	1,138 (19.1)	1,132 (20.1)	1,176 (20.4)	964 (19.9)
	Middle-high	1,169 (19.3)	1,102 (18.2)	1,085 (20.2)	1,055 (19.6)	1,053 (20.0)	1,084 (19.6)	1,163 (21.0)	1,140 (19.8)	1,150 (20.3)	927 (20.0)
	High	1,153 (17.7)	1,086 (18.0)	1,060 (19.3)	1,076 (20.2)	1,060 (20.0)	1,102 (20.7)	1,103 (19.8)	1,137 (18.9)	1,145 (20.3)	967 (21.4)

Values are presented as number (weighted %).

1 Calculated as the monthly household income divided by square-root of the number of persons in the household, categorized into quintiles according to age and gender.

**Table 2.** Trends in dietary habits in Korean men and women aged 19 years or older using data from the 2011-2020 Korea National Health and Nutrition Examination Survey

Variables	Category	2011	2012	2013	2014	2015	2016	2017	2018	2019	2020	Trend^1^				Difference^1^	
												2011-2020		2011-2019		2019-2020	
												β estimate	p-value	β estimate	p-value	Difference	p-value
Skipping breakfast (%)^2^	Total	22.5	24.6	25.2	25.6	28.0	29.6	29.8	30.9	34.1	36.7	0.013	<0.001	0.012	<0.001	2.6	0.080
	Men	22.9	23.5	27.2	27.5	29.5	32.4	33.0	30.6	35.4	38.8	0.015	<0.001	0.014	<0.001	3.4	0.081
	Women	22.0	25.7	22.9	23.5	26.2	26.4	26.4	31.2	32.6	34.3	0.012	<0.001	0.010	<0.001	1.7	0.343
Eating out more than once a day (%)^3^	Total	29.4	25.6	31.9	30.6	31.3	32.3	30.1	33.5	31.0	26.4	0.001	0.313	0.004	<0.001	-4.6	0.001
	Men	41.6	36.5	45.2	43.3	43.1	45.8	41.2	44.8	41.0	35.8	-0.002	0.119	0.002	0.325	-5.2	0.012
	Women	16.8	14.4	18.1	17.3	19.2	18.3	18.4	21.8	20.5	16.7	0.004	<0.001	0.006	<0.001	-3.8	0.014
Dinner with family members (%)^4^	Total	62.7	63.0	61.3	61.6	60.6	60.5	61.3	61.3	64.5	65.1	0.001	0.376	-0.001	0.673	0.6	0.735
	Men	58.9	62.6	57.7	57.0	58.1	56.2	57.6	58.3	60.4	63.3	0.001	0.507	-0.002	0.402	2.9	0.198
	Women	67.2	63.9	65.6	67.0	63.6	65.6	65.7	65.0	69.4	67.4	0.001	0.451	0.000	0.761	-2.0	0.271
Household with food security (%)^5^	Total	95.2	92.2	93.5	93.8	93.5	95.8	96.3	96.9	96.5	96.3	0.002	0.002	0.002	0.003	-0.2	0.658

Values are presented as the weighted %; Age-standardized prevalence was calculated using the 2005 Korean Census.

1 Values for trends and differences are adjusted for age and household income.

2 The proportion of population skipping breakfast 1 day before the survey.

3 The proportion of population eating out more than once a day.

4 The proportion of the population who ate dinner with family members.

5 The proportion of households that responded “all members of our family were able to eat enough amount and various kinds of foods as much as we need” or “all members of our family were able to eat enough amount, but we were not able to eat various kinds of foods.”

**Table 3.** Trends in the intakes of major food groups among Korean men and women aged 19 years or older using data from the 2011-2020 Korea National Health and Nutrition Examination Survey

Variables	Category	2011	2012	2013	2014	2015	2016	2017	2018	2019	2020	Trend^1^				Difference^1^	
												2011-2020		2011-2019		2019-2020	
												β estimate	p-value	β estimate	p-value	Difference	p-value
Grains (g/day)	Total	312.3	304.4	303.3	296.0	304.4	295.9	290.1	293.4	274.4	271.0	-4.221	<0.001	-3.768	<0.001	-3.4	0.468
	Men	350.8	342.3	337.8	332.6	346.9	335.0	325.2	335.6	314.2	310.0	-3.857	<0.001	-3.300	<0.001	-4.3	0.531
	Women	273.5	266.3	268.1	258.7	261.2	255.9	254.4	249.7	233.4	230.9	-4.736	<0.001	-4.412	<0.001	-2.5	0.636
Vegetables (g/day)	Total	328.2	322.9	320.0	328.4	317.8	293.8	299.8	275.8	283.7	276.2	-6.086	<0.001	-6.167	<0.001	-7.5	0.187
	Men	376.7	361.5	361.7	365.3	361.5	335.5	338.8	315.9	323.8	312.8	-7.017	<0.001	-6.945	<0.001	-10.9	0.172
	Women	279.7	284.8	278.5	291.4	273.4	252.2	260.1	235.2	242.5	239.1	-5.371	<0.001	-5.622	<0.001	-3.4	0.542
Fruits (g/day)	Total	178.0	175.5	175.0	188.2	200.2	176.0	156.4	134.5	140.6	124.9	-5.928	<0.001	-4.787	<0.001	-15.8	0.054
	Men	163.8	161.0	156.7	161.4	181.4	163.6	139.1	120.5	124.4	111.4	-5.841	<0.001	-4.872	<0.001	-13.1	0.116
	Women	192.7	190.2	194.4	215.9	219.6	189.2	174.1	149.1	157.3	139.0	-5.951	<0.001	-4.630	<0.001	-18.3	0.094
Meat (g/day)	Total	109.6	115.0	104.9	105.6	109.8	114.4	117.7	118.9	125.2	128.4	1.962	<0.001	1.616	<0.001	3.3	0.489
	Men	136.3	146.4	130.6	128.2	137.2	145.9	149.4	149.3	159.2	165.2	2.924	<0.001	2.376	<0.001	6.0	0.420
	Women	82.5	82.6	78.3	82.3	81.3	81.0	84.1	86.6	88.9	89.6	0.921	0.002	0.743	0.032	0.7	0.861
Non-alcoholic beverages (g/day)	Total	129.6	141.0	186.2	192.2	210.9	231.8	229.0	230.6	247.0	254.3	12.394	<0.001	13.247	<0.001	7.3	0.517
	Men	141.3	152.7	206.7	214.8	240.8	263.4	252.2	249.0	272.6	277.6	13.768	<0.001	14.751	<0.001	5.0	0.771
	Women	118.0	129.2	165.0	168.3	179.2	197.8	204.3	210.2	219.5	230.0	10.988	<0.001	11.678	<0.001	10.5	0.345

Values are presented as the weighted mean intake (g/day); Age-standardized mean was calculated using the 2005 Korean Census.

1 Values for trends and differences are adjusted for age and household income.

**Table 4.** Trends in energy intake among Korean men and women aged 19 years or older using data from the 2011-2020 Korea National Health and Nutrition Examination Survey

Variables		Category	2011	2012	2013	2014	2015	2016	2017	2018	2019	2020	Trend^1^				Difference^1^	
													2011-2020		2011-2019		2019-2020	
													β estimate	p-value	β estimate	p-value	Difference	p-value
Energy (kcal)		Total	2,090.9	2,057.1	2,135.7	2,121.7	2,174.8	2,112.4	2,071.8	2,042.6	1993.0	1,953.4	-14.505	<0.001	-9.862	<0.001	-39.6	0.159
		Men	2,464.3	2,412.5	2,477.5	2,454.6	2,538.7	2,491.1	2408.0	2,398.2	2,338.1	2,281.4	-17.512	<0.001	-11.702	0.001	-56.7	0.175
		Women	1,715.4	1,698.5	1,787.2	1,781.7	1,803.7	1,722.1	1,724.7	1,673.6	1,634.4	1,614.7	-12.987	<0.001	-9.771	<0.001	-19.7	0.464
Energy-source nutrient^2^																		
	Fat (%)	Total	19.2	19.8	20.4	21.0	21.1	21.2	21.5	22.0	23.1	24.1	0.477	<0.001	0.417	<0.001	1.0	0.002
		Men	19.9	20.5	20.9	21.5	21.5	21.8	21.8	22.3	23.4	24.3	0.417	<0.001	0.354	<0.001	0.9	0.026
		Women	18.6	19.1	19.9	20.5	20.7	20.6	21.2	21.7	22.7	23.8	0.533	<0.001	0.477	<0.001	1.0	0.003
	Protein (%)	Total	14.9	14.9	14.6	14.7	14.7	15.3	15.4	15.5	15.9	15.9	0.133	<0.001	0.126	<0.001	0.0	0.939
		Men	15.4	15.4	15.2	15.1	15.2	15.9	15.9	16.0	16.3	16.5	0.131	<0.001	0.120	<0.001	0.2	0.499
		Women	14.3	14.5	14.0	14.2	14.2	14.7	14.8	15.0	15.4	15.2	0.132	<0.001	0.128	<0.001	-0.1	0.449
	Carbohydrates (%)	Total	65.9	65.3	65.0	64.3	64.2	63.5	63.1	62.5	61.1	60.1	-0.609	<0.001	-0.543	<0.001	-1.0	0.008
		Men	64.7	64.2	63.8	63.3	63.4	62.3	62.2	61.7	60.3	59.2	-0.548	<0.001	-0.474	<0.001	-1.1	0.021
		Women	67.1	66.4	66.0	65.2	65.1	64.7	64.0	63.3	61.9	61.0	-0.665	<0.001	-0.605	<0.001	-0.9	0.047
Meal type																		
	Home-cooked meal (%)	Total	-	-	-	-	-	39.7	39.6	39.2	37.5	39.9	-0.207	0.308	-0.786	0.004	2.5	0.011
		Men	-	-	-	-	-	35.9	35.8	36.2	34.6	37.0	0.065	0.807	-0.357	0.314	2.5	0.057
		Women	-	-	-	-	-	43.7	43.6	42.3	40.4	42.9	-0.456	0.052	-1.171	<0.001	2.6	0.019
	Restaurant meal (%)	Total	-	-	-	-	-	31.8	33.0	32.5	33.5	31.9	0.109	0.587	0.478	0.072	-1.5	0.180
		Men	-	-	-	-	-	36.4	37.2	35.7	36.7	35.1	-0.220	0.436	-0.040	0.917	-1.5	0.282
		Women	-	-	-	-	-	27.0	28.7	29.2	30.3	28.7	0.420	0.056	0.965	0.001	-1.4	0.243
	Food services meal at schools or workplace (%)	Total	-	-	-	-	-	5.2	5.2	5.6	5.8	3.8	-0.222	0.021	0.212	0.132	-2.0	<0.001
		Men	-	-	-	-	-	6.7	6.3	6.7	7.2	4.5	-0.350	0.019	0.183	0.417	-2.8	<0.001
		Women	-	-	-	-	-	3.5	4.0	4.5	4.2	3.1	-0.105	0.222	0.217	0.069	-1.1	0.013
	Convenience food (%)	Total	-	-	-	-	-	7.3	7.4	8.3	8.5	9.7	0.591	<0.001	0.434	<0.001	1.2	0.014
		Men	-	-	-	-	-	7.8	7.9	8.8	8.9	10.3	0.617	<0.001	0.474	0.006	1.4	0.052
		Women	-	-	-	-	-	6.9	7.0	7.6	8.1	9.1	0.562	<0.001	0.393	0.005	1.1	0.069

Values are presented as the weighted mean intake or weighted %; Age-standardized mean was calculated using the 2005 Korean Census.

1 Values for trends and differences are adjusted for age and household income. For meal type, trends were calculated using 2016-2020 and 2016-2019 data, respectively.

2 2The percentage of energy from fat refers to the percentage of energy from fat (g of fat×9 kcal/g) compared with the sum of energy from fat, energy from carbohydrates, and energy from protein. The respective percentages of energy from the other components were calculated using a similar equation.

**Table 5.** Trends in nutrient intakes among Korean men and women aged 19 years or older using data from the 2011-2020 Korea National Health and Nutrition Examination Survey

Variables	Category	2011	2012	2013	2014	2015	2016	2017	2018	2019	2020	Trend^1^				Difference^1^	
												2011-2020		2011-2019		2019-2020	
												β estimate	p-value	β estimate	p-value	Difference	p-value
Calcium (mg/d)	Total	518.0	508.3	500.3	494.6	510.1	526.5	524.4	517.3	503.6	493.9	0.145	0.830	1.349	0.088	-9.7	0.246
	Men	581.1	546.2	552.6	544.7	568.1	587.6	569.6	580.2	550.3	536.8	-0.642	0.502	1.317	0.240	-13.5	0.243
	Women	454.6	470.7	448.4	443.8	451.3	464.7	478.7	453.0	456.3	450.0	0.677	0.368	1.100	0.220	-6.3	0.522
Sodium (mg/d)	Total	5,211.0	4,942.1	4,176.3	4,033.2	4,188.7	3,585.5	3,586.5	3,488.4	3,512.3	3,412.6	-184.720	<0.001	-207.719	<0.001	-99.7	0.103
	Men	6,177.9	5,726.9	4,891.0	4,698.1	4,984.6	4,244.5	4,171.1	4,133.5	4,164.6	4,037.5	-216.195	<0.001	-242.554	<0.001	-127.2	0.134
	Women	4,241.6	4,154.3	3,448.3	3,354.2	3,379.9	2,910.4	2,989.8	2,820.8	2,836.0	2,769.3	-156.859	<0.001	-177.062	<0.001	-66.7	0.260
Iron (mg/d)	Total	15.4	15.3	18.2	17.9	17.8	12.5	12.2	12.1	11.8	11.2	-0.718	<0.001	-0.712	<0.001	-0.6	0.003
	Men	17.5	16.9	21.0	20.4	20.2	14.1	13.7	13.9	13.6	12.9	-0.801	<0.001	-0.793	<0.001	-0.7	0.026
	Women	13.2	13.6	15.3	15.4	15.3	10.7	10.7	10.2	10.0	9.5	-0.644	<0.001	-0.642	<0.001	-0.6	0.011
Vitamin A (μgRAE/d)	Total	-	-	-	-	-	403.4	381.7	383.6	379.9	396.0	-1.035	0.745	-4.865	0.237	16.1	0.207
	Men	-	-	-	-	-	444.6	406.3	420.1	410.6	418.4	-5.728	0.157	-8.334	0.116	7.8	0.643
	Women	-	-	-	-	-	362.1	357.3	346.5	348.4	373.0	3.461	0.360	-1.809	0.724	24.6	0.065
Riboflavin (μg/d)	Total	1,297.7	1,312.3	1,415.0	1,443.7	1,466.4	1,657.3	1,645.5	1,667.9	1,694.4	1,756.4	52.222	<0.001	53.704	<0.001	62.0	0.040
	Men	1,493.7	1,486.1	1,600.3	1,643.7	1,655.1	1,907.2	1,860.8	1,909.4	1,916.2	2,009.1	59.071	<0.001	60.475	<0.001	92.9	0.031
	Women	1,105.0	1,136.8	1,227.5	1,237.5	1,274.7	1,398.4	1,424.1	1,416.9	1,463.5	1,494.1	44.641	<0.001	46.019	<0.001	30.5	0.300
Vitamin C (mg/d)	Total	109.2	110.0	95.2	101.8	100.7	62.8	63.5	61.3	67.0	64.2	-6.408	<0.001	-7.193	<0.001	-2.8	0.302
	Men	115.2	114.0	92.0	96.5	97.8	63.0	64.2	67.6	67.7	68.0	-6.310	<0.001	-7.248	<0.001	0.3	0.936
	Women	103.3	106.2	98.6	107.3	103.7	62.8	62.7	54.7	66.1	60.2	-6.510	<0.001	-7.143	<0.001	-5.9	0.040

Values are presented as the weighted mean intake; Age-standardized mean was calculated using the 2005 Korean Census.

1 Values for trends and differences are adjusted for age and household income. For vitamin A, trends were calculated using 2016-2020 and 2016-2019 data, respectively.

## SUPPLEMENTARY MATERIALS

Korean version is available at https://www.e-epih.org/.
