# Supplementary material for: Changes in food and nutrient intakes in Korean adults before and during the COVID-19 pandemic: data from the 2011-2020 Korea National Health and Nutrition Examination Survey
Source: Epidemiol Health. 2023 Feb 1;45:e2023015. doi: 10.4178/epih.e2023015 (PMC10581887; doi:10.4178/epih.e2023015)
Supplement: Supplementary file 1 [file epih-45-e2023015-Korean-Supplementary.docx]

**ABSTRACT**

**목적:** 본 연구는 국민건강영양조사를 통해 2011년부터 2020년까지 최근 10년(2011년-2020년) 및 코로나19 유행 전(2019년)·후(2020년)의 식생활, 식품 및 영양소 섭취 변화를 파악하고자 한다.

**방법:** 연구대상은 2011년부터 2020년 국민건강영양조사 영양조사에 참여한 만19세이상, 총 54,995명이었다. 최근 10년간(2011-2020년) 식생활, 식품 및 영양소 섭취량 또는 분율을 산출하였고, 추이와 코로나19 유행 전(2019년)과 후(2020년)의 차이는 PROC SURVEYREG를 이용하여 통계적 유의성을 검정하였다.

**결과:** 최근 10년간(2011년-2020년) 성인의 식생활(결식, 외식 증가), 식품(채소류, 과일류 섭취 감소, 음료류, 육류 섭취 증가 등) 및 영양소(지방 섭취 증가, 나트륨, 비타민 C 섭취 감소 등) 섭취가 변화하였다. 코로나19 유행 전(2019년)·후(2020년)를 비교 시, 2020년 하루 1회이상 외식률은 26.4%로 2019년(31.0%)에 비해 4.6%p 감소하여 10년간 추이와는 다른 양상이었다. 코로나19 유행 전(2019년)과 후(2020년)의 식품, 에너지, 대부분의 영양소 섭취는 통계적으로 유의한 수준의 차이가 없었으며, 에너지에서 차지하는 지방, 탄수화물 섭취 분율은 각각 1%p 증가, 감소하였다.

**결론:** 코로나19 유행 전, 후 비교 시, 외식 감소, 가정식 섭취 증가 등 식생활은 변화가 있었으나 주요 식품 및 영양소 섭취는 큰 변화없이 최근 10년간의 추이가 지속되고 있었다. 코로나19 유행이 지속되는 상황을 고려하여 식생활 변화가 식품 및 영양소 섭취, 건강 수준에 미치는 영향에 관한 모니터링이 필요하다.

**주제어: 코로나19 유행, 국민건강영양조사, 식생활, 식품, 영양소**

**서론**

2020년에 발생한 코로나19 유행으로 인한 사회적 거리두기는 일상생활 습관의 변화와 함께 가구 또는 개인의 식품 선택과 구매 방법, 식습관, 식사의 양과 질, 식품 및 영양소 섭취에 영향을 미쳤다 [1-3]. 개인의 영양 불균형은 면역기능을 저하시켜 코로나19 감염 및 중증 위험을 증가시키고, 비만, 당뇨병, 고혈압 등 만성질환의 증가와 관련이 있다 [4,5]. 만성질환 또한 코로나19 감염 및 중증의 위험요인이므로 [6], 만성질환 뿐만 아니라 감염성질환 예방 및 관리에서의 영양의 중요성을 고려하여 세계보건기구 등 국제기구와 각 나라는 코로나19 감염 예방을 위해 적절한 양의 물 섭취, 과일·채소류, 좋은 급원의 단백질 섭취 등 건강한 식습관 유지를 권고하였다 [7].

코로나19 유행 이후, 다양한 연구대상 및 방법으로 식습관 및 영양상태 변화에 관한 연구가 광범위하게 수행되었으며, 상반된 방향의 연구결과가 발표되고 있다. 코로나19 유행 이후 식사 및 간식 횟수 증가, 과일·채소류 섭취 감소, 가공식품, 당류 섭취 증가 등 식생활이 악화되었다는 연구결과가 보고된 반면 [8-13], 일부 연구 [14,15]에서는 코로나19 감염 예방 및 회복을 고려하여 과일·채소류 섭취 증가, 식사의 질 향상 등 식생활이 개선되었고, 한 연구내에서도 연령, 소득수준, 건강행태 등 개인별 특성에 따라 식습관이 개선 또는 악화되는 방향의 변화가 있었다 [8,16,17]. 현재까지 수행된 대부분의 연구가 편의표본 대상의 간단한 설문을 이용한 온라인방법으로 수행되었고, 몇몇 타당도가 확보된 영양조사 방법을 적용한 코호트 연구는 표본 규모가 적은 제한점이 있다 [3].

국내에서는 코로나19 유행 이후 청소년에서 결식 증가, 외식 감소, 과일류 섭취 감소 등 식습관 변화와 신체활동 감소가 보고된 바 있다 [18]. 성인에서도 코로나19 유행 이후 신체활동은 감소하고 비만, 고콜레스테롤혈증 등 만성질환 유병이 큰 폭으로 증가하였고 특히 이러한 경향은 남자에서 뚜렷하였다 [19,20]. 식생활 측면에서는 코로나 19 유행 이후 배달음식, 인스턴트 음식 섭취 증가한 비율이 각각 38.5%, 21.5%로 보고된 바 있으나 [21], 식품 및 영양소 섭취 수준 변화에 관한 체계적인 연구결과는 발표된 바 없다.

본 연구는 국민건강영양조사를 통해 2011년부터 2020년까지 최근 10년간의 식품 및 영양소 섭취 추이 및 코로나19 유행 전(2019년)·후(2020년)의 식생활, 식품 및 영양소 섭취 변화를 파악하기 위해 수행하였다.

**연구방법**

**조사대상**

국민건강영양조사는 1998년부터 국민의 건강 및 영양상태를 파악하기 위해 질병관리청이 매년 실시하는 국가단위의 건강조사이다. 우리 국민을 대표할 수 있도록 2단계 층화집락표본 추출방법을 이용하여 연간 약 200개 조사구, 조사구당 20~25개 가구의 표본을 추출하였다. 추출틀은 인구주택총조사 자료이며, 층화변수는 시도, 동읍면, 주택유형(일반/아파트)을 적용하였고, 내재적 층화변수로 주거면적, 가구주 학력 등을 추가로 고려하여 변화된 모집단 특성을 반영함과 동시에 표본의 대표성과 시계열적 일관성 유지가 가능하도록 하였다 [22]. 조사대상은 위의 방법을 이용하여 추출된 표본가구 내 만1세 이상의 모든 가구원이었고, 본 연구의 분석 대상은 2011년부터 2020년 국민건강영양조사 영양조사를 완료한 만19세이상, 총 54,995명이었다. 2011년-2019년은 표본으로 추출한 모든 조사구에 대한 조사를 완료하였으나 2020년은 코로나19 유행으로 인해 192개 조사구 중 166개에 대한 조사(86%)를 완료하였다. 2020년 연구대상자의 성별, 연령별, 소득수준별 특성을 2019년과 비교 시 통계적으로 유의한 차이가 없었다.

**조사방법**

국민건강영양조사는 검진조사, 건강설문조사, 영양조사로 구성되어 있다. 영양조사는 식생활조사와 식품섭취조사로 구분되며, 조사팀(영양사)이 조사 대상자의 가구를 방문하여 개인별 면접방법으로 실시하였다. 식생활조사는 결식, 외식, 동반식사 여부 등에 관하여 설문을 이용하여 조사하고, 식품섭취조사는 24시간 회상방법으로 조사일 하루 전날 섭취한 모든 음식에 대해서 시간 순서대로 식사 장소, 매식 종류와 함께 음식 종류와 섭취량을 조사하였다. 정확한 섭취량 파악을 위해 2차원 모델집, 계량컵, 계량스푼 등 보조도구를 이용하여 개인별 섭취량을 조사하였고, 에너지 및 영양소 섭취량은 국가표준식품성분표를 기반으로 구축한 식품별 영양성분 DB를 활용하여 산출하였다 [23].

식생활조사를 통해 아침식사 결식률, 하루 1회이상 외식률, 저녁식사 가족동반 식사율, 식품안정성 확보 가구분율을 산출하였고, 식품섭취조사를 통해 식품, 에너지 및 영양소 섭취를 산출하였다. 본 연구에서 식품은 연도별 섭취량 변화가 큰 곡류, 채소류, 과일류, 육류, 음료류를, 영양소는 제4차 국민건강증진종합계획 2020-2030(Health Plan 2030) 영양 중점과제 성과지표의 구성요소인 에너지, 지방, 단백질, 탄수화물, 칼슘, 나트륨, 철, 비타민 A, 리보플라빈, 비타민 C를 중점적으로 검토하였다. 비타민 A는 2015 한국인 영양소 섭취기준의 비타민 A 기준 단위가 ㎍ RAE로 변경됨에 따라 2016년부터 산출하였다. 에너지 구성의 변화를 검토하기 위해 에너지 영양소별(지방, 탄수화물, 단백질), 음식 종류별(가정식, 음식업소 음식, 단체급식, 편의식품) 에너지 섭취 분율을 산출하였다. 음식 종류는 가정식, 음식업소 음식(음식업소에서 먹거나, 음식을 포장, 배달하여 음식업소가 아닌 장소에서 먹은 경우를 포함), 단체급식, 편의식품(즉석조리식품, 반조리식품 등)을 같은 기준으로 분류하기 시작한 2016년부터 산출하였다.

**분석방법**

자료의 분석은 SAS version 9.4 (SAS Institute Inc., Cary, N.C.)를 이용하였으며, 모든 결과는 목표 모집단인 우리나라에 거주하는 국민의 특성을 잘 대표할 수 있도록 가중치를 적용하여 복합표본설계분석방법으로 산출하였다. 식생활, 식품 및 영양소에 따른 연도별 섭취량 또는 분율은 연령구조 차이에 따른 영향을 보정하기 위해 2005년 추계인구로 표준화하였고, PROC SURVEYREG, PROC SURVEYLOGISTIC을 이용하여 계산하였다. 최근 10년간(2011-2020년)과 2011-2019년의 식생활, 식품 및 영양소 섭취 추이 분석은 연령, 소득수준을 보정하여 산출하였으며, 추이 분석 및 코로나19 유행 전(2019년)과 후(2020년)의 차이 비교에 대한 통계적 유의성 검정은 PROC SURVEYREG를 이용하여 계산하였다.

**윤리 성명**

본 연구는 질병관리청 연구윤리심의위원회를 통해서 연도별 계획에 따라 승인을 받았으며( 2011-2014년, 2018년-2020년), 일부 연도(2015-2017년)는 생명윤리법 제2조제1호 및 동 시행규칙 제2조제2항제1호에 따라 심의면제를 받았다.

**연구결과**

본 연구대상자는 2011년-2020년 국민건강영양조사 중 영양조사를 완료한 총 54,995명이었으며 남자 23,137명, 여자 31,858명이었다. 성별, 연령별, 소득수준별 분포는 표 1에 제시하였다.

최근 10년간(2011-2020년) 식생활 추이에서, 아침식사 결식률은 2011년 22.5%에서 지속적으로 증가하여 2020년 36.7%였다(표 2). 하루 1회이상 외식률은 여자에서만 증가 경향이었고, 식품안정성 확보가구 분율은 증가하는 경향으로 2020년 96.3%였다. 코로나19 유행 전·후를 비교하면, 2020년 아침식사 결식률은 36.7%로, 2019년(34.1%)에 비해 2.6%p 증가하였으나 통계적으로 유의한 수준은 아니었다. 2020년 하루 1회이상 외식률은 26.4%로 2019년(31.0%)에 비해 4.6%p(남자 5.2%p, 여자 3.8%p) 감소하여 10년간 추이와는 다른 양상이었고, 식품안정성 확보가구 분율은 2019년과 2020년이 차이가 없었다.

주요 식품 섭취의 경우 최근 10년간 곡류, 채소류, 과일류의 섭취는 감소 경향이었고, 육류와 음료류 섭취는 증가 경향이었다(표 3). 특히, 과일류는 2011년 178g에서 2020년 125g으로 약 50g 감소하였고, 음료류는 2011년 130g에서 2020년 254g으로 2배 정도 증가하였으며 남녀모두 같은 경향이었다. 코로나19 유행 전·후를 비교하면, 주요 식품군 모두 최근 10년 추이와 유사하게 2019년에 비해 2020년의 곡류, 과일류, 채소류의 섭취는 감소하고 음료류와 육류의 섭취는 증가하였으나 통계적으로 유의한 수준의 차이는 아니었다.

에너지 섭취는 최근 10년간 남자의 경우 약 200kcal, 여자의 경우 약 100kcal 감소하였다(표 4). 에너지 급원별로 구분 시, 지방 섭취분율은 지속 증가하여 2020년 24.1%, 탄수화물 섭취분율은 감소 경향으로 2020년 60.1%였다. 코로나19 유행 전·후를 비교 시, 2020년 에너지 섭취는 1,953kcal로, 2019년과 비교 시 약 40kcal(남자 57kcal, 여자 20kcal) 감소하였으나 통계적으로 유의한 수준은 아니었다. 2020년 지방 섭취분율은 24.1%로 19년에 비해 1.0%p 증가, 2020년 탄수화물 섭취분율은 60.1%로 2019년에 비해 1.0%p 감소하여 10년간 추이와 같은 경향이었다. 최근 5년간 에너지 섭취에서 차지하는 가정식(여자)과 단체급식(남자) 분율은 감소 경향이었고 음식업소(여자)와 편의식품(남, 여 모두 해당) 분율은 증가 경향이었다. 코로나19 유행 전·후를 비교 시, 2020년 에너지 섭취에서 차지하는 가정식, 음식업소, 단체급식, 편의식품 분율은 각각 39.9%, 31.9%, 3.8%, 9.7%로, 2016-2020년 추이와는 다르게 2019년에 비해 가정식 분율은 증가(2.5%p) 하였고, 음식업소 음식 분율은 감소하였으나 통계적으로 유의한 수준의 차이는 아니었다. 2020년 단체급식과 편의식품 분율은 2019년에 비해 각각 감소(2.0%p), 증가(1.2%p)하여 2016-2020년 추이와 같은 방향이었다.

최근 10년간 나트륨, 철, 비타민 C 섭취는 지속적인 감소 경향, 리보플라빈의 섭취는 증가 경향이었다(표 5). 코로나19 유행 전·후를 비교 시, 2019년에 비해 2020년 비타민 C(여자)와 철(남, 여 모두 해당) 은 감소, 리보플라빈(남자)은 증가하여 10년간의 추이가 지속되었고, 칼슘, 나트륨, 비타민 A 섭취는 남, 여 모두 2019년과 2020년이 차이가 없었다.

**고찰**

최근 10년간(2011년-2020년) 성인의 식품 및 영양소 섭취를 분석한 결과, 식생활(결식, 외식 증가), 식품(채소류, 과일류 섭취 감소, 음료류, 육류 섭취 증가 등), 영양소(지방 증가, 나트륨, 비타민 C 감소 등) 섭취 추이는 선행연구 [23]에서 발표한 약 20년간(1998년-2020년) 추이와 크게 다르지 않았다. 이는 가구구조 변화, 식품가공 기술 발전, 외식 종류 다양화 등 식생활과 관련된 사회환경의 점진적인 변화에 의한 것으로 여겨진다. 다른 나라도 경제 및 인구구조 변화로 인한 소득, 도시화, 식품산업 발전, 영양정책 변화에 따라 영양 추이 변화(nutrition transition)가 있었다 [24]. 일본의 경우 우리나라와 유사하게 2000년대 초반 이후 지방 섭취가 지속적으로 증가하여 2019년 기준 28.6%이었고 나트륨 섭취는 감소 경향이었다 [25, 26]. 유럽은 최근 30년간 과일류, 채소류, 생선류 섭취 증가, 포화지방산, 당류 섭취 감소 등 바람직한 방향으로 변화하였고 [27], 미국도 여전히 포화지방산과 단순당의 섭취가 높은 영양문제는 있으나 2000년 이후 양질의 탄수화물, 식물성 단백질, 다불포화지방산 섭취 증가 등 식생활의 질이 전반적으로 개선되고 있다 [28]. 이와는 달리, 우리나라의 경우 국민건강영양조사를 실시한 1998년 이후 나트륨 섭취가 개선된 점을 제외하고서는 대부분의 식생활 및 영양소 섭취 추이가 악화되어 이를 전환하기 위한 영양 개입을 적극적으로 추진할 시점이라고 여겨진다.

코로나19 유행 전(2019년), 후(2020년) 성인의 식생활을 비교 시, 가정식과 편의식품 섭취는 증가하고 외식이 감소하는 등 변화가 있었다. 이는 근무형태 변화, 사적모임 및 다중이용시설 제한 등 사회적 거리두기에 의한 것으로 보이며, 국내·외 다른 연구결과와 유사하였다. 2020년 식품소비행태조사 결과 [29], 성인의 외식 비율은 2019년에 비해 7.5%p 감소하였고 이 경향은 2021년에도 지속되었고 [30], 국외 연구 [10,31]에서도 코로나19 유행함에 따라 외식은 감소하고 가정식 및 가공식품 섭취가 증가하였다. 또한, 국외연구에서 사회적 거리두기 시기에 배달음식 섭취가 증가하였고 [10], 2020년 지역사회건강조사[21]에서도 배달음식 섭취가 늘었다는 비율(38.5%)이 줄었다는 비율(11.2%)에 비해 높았다. 이를 고려하여 본 연구에서 음식업소음식을 식사장소에 따라 분류하여 에너지 섭취에서 차지하는 분율을 비교하였다. 2019년에 비해 2020년 음식업소에서 먹는 분율(2019년 23.6%, 2020년 20.3%)은 3.3%p 감소하였으나 가정에서 배달, 포장을 통한 음식업소음식을 섭취하는 분율(2019년 6.5%, 2020년 8.6%)은 2.1%p 증가하여(data not shown), 선행 연구결과와 유사하였다 [10,21].

이 외에도 코로나19 유행에 따른 식생활 변화 양상에 관한 다양한 연구결과가 발표되고 있다. 코로나19 유행 이후 식사 및 간식 [9,12], 야식 횟수 증가 [9], 에너지 밀도가 높은 음식의 섭취 증가 [11] 등 전반적인 식습관 및 식사의 질 악화 [13,32], 이로 인한 체중 증가 [10,12] 등 바람직하지 않는 방향의 변화가 있었다. 반면, 다른 연구 [14,15]에서는 감염 예방을 위한 항산화기능, 항염증 및 면역기능 향상을 고려하여 당류, 패스트푸드 섭취 감소 등 긍정적인 방향으로 변화하였다. 이러한 상반된 연구결과는 코로나19 유행 심각도를 고려한 사회적 거리두기 단계 [13,33], 거주 지역 [16], 가구 특성, 소득, 신체활동 등 개인의 건강행태 [17] 차이에서 기인한 것으로, 우리나라의 코로나19 유행 특성, 사회경제위치, 건강행태 수준에 따른 식생활 및 식사의 질 변화에 대한 심층분석을 추가로 수행할 필요가 있다.

본 연구에서 코로나19 유행 전(2019년), 후(2020년) 식품, 에너지 및 영양소 섭취는 최근 10년간(2011-2020년) 추이와 큰 차이가 없었다. 2020년 음식종류별 에너지 섭취에서 차지하는 분율을 산출하여 2019년과 비교 시, 음식업소음식(-1.5%p)과 단체급식은 감소(-2.0%p)한 반면 가정식은 증가(2.5%p)하여 음식종류별 급원은 변화가 있었으나 이 변화 정도가 크지 않아 2020년 식품 및 영양소 섭취에 큰 영향을 미치지 않았을 가능성이 있다. 에너지 급원별 분율에서는 2019년에 비해 2020년 지방의 섭취분율은 증가, 탄수화물 섭취분율은 감소하였다. 2020년 지방의 절대 섭취량은 52.6g으로 2019년(51.1g)에 비해 1.5g 증가하였으나 유의미한 변화가 아니어서(p=0.23) 지방 섭취분율은 에너지 섭취 감소(약 40kcal)로 인해 차이가 있을 가능성이 있다. 또한, 2019년에 비해 2020년의 비타민 C(특히, 여자) 감소와 리보플라빈(특히, 남자) 증가는 과일류(여자, 18.3g 감소, p=0.09)와 육류 섭취(남자, 6.0g 증가, p=0.42) 경향을 일부 반영한 것으로 보인다. 추가적으로, 2019년과 2020년의 나트륨, 칼슘, 비타민 A 적정섭취, 에너지/지방 과잉 섭취, 영양소 섭취 부족 분율을 비교한 결과 유의한 수준의 차이는 없었다(data not shown). 24시간회상법, 식사기록법, 식품섭취빈도조사 등을 활용한 국외 연구결과도 본 연구의 결과와 유사하였다. 영국 영양조사(National Diet and Nutrition Survey) [34]의 경우 2019년에 비해 2020년의 에너지 섭취는 다소 감소하였으나 식품 및 영양소의 섭취는 차이가 없었다. 건강한 폐경후 여자를 대상으로 스페인에서 수행한 연구에서도 에너지 및 영양소 섭취 모두 차이가 없었고[35], 성인 대상으로 미국에서 수행한 연구에서도 에너지 밀도는 증가했지만 에너지 섭취는 차이가 없었다[11]. 독일 어린이와 청소년에서는 코로나19 유행 전에 비해 가당음료 및 초가공식품의 섭취가 다소 감소하였으나 에너지 섭취(0.85% 감소)는 큰 변화가 없었다[36].

우리나라의 식품, 영양소 섭취가 코로나19 유행임에도 전반적으로 큰 폭으로 악화되지 않은 채 유지되고 있는 상황은 긍정적인 결과임이 분명하다. 그러나, 본 연구결과는 코로나19 유행이 시작한 첫 해의 결과이며 코로나19 유행이 지속되는 상황을 고려하면, 식생활의 변화가 독립적으로 또는 음주 등 다른 생활습관과 상호작용하여 식품 및 영양소 섭취에 유의한 수준의 변화를 유발, 만성질환 증가에 기여할 가능성이 있다. 청소년건강행태조사에서 코로나19 유행 첫 해인 2020년에 신체활동이 감소한 후 2021년에는 다소 증가한 반면 결식, 과일·채소류 섭취 등 식생활은 2021년에도 지속적으로 악화된 점은 시사하는 바가 있다 [18]. 코로나 19 유행 2년차(2021년), 3년차(2022년)의 식생활 변화 지속 여부, 이러한 변화가 식품 및 영양소 섭취에 영향 여부에 관한 모니터링과 추이 악화를 예방하기 위한 영양 개입의 근거 마련이 필요하다. 국민건강영양조사는 조사가 시작된 1998년 이후 동일한 방법(24시간 회상조사)으로 지침 교육을 이수한 조사원(영양사)에 의해 수행하고 있다. 또한, 본 연구에서 식품 및 영양소 섭취량 산출 시, 가용한 최신의 음식별 식품재료량 DB, 식품별 영양성분 DB를 적용하고 있다. 즉, 2011-2012년, 2013-2015년, 2016-2020년 식품 및 영양소 섭취량 산출 시 해당년도별로 최신의 국가표준식품성분표인 제7개정판, 제8개정판, 제9개정판을 각각 활용하였다. 영양상태를 평가하는 시점의 음식 정보 또는 식품의 영양성분 정보를 시의성있게 반영한다는 점에서는 장점이지만 식품 및 영양소 섭취량 추이 비교 시 적용된 DB의 영향을 고려해야 하는 제한점이 있다.

**결론**

국민건강영양조사 자료를 이용하여 최근 10년간(2011-2020년) 성인의 식품 및 영양소 섭취 추이를 분석한 결과, 육류, 음료류, 지방 섭취가 증가하고 곡류, 채소·과일류, 비타민 C, 나트륨 섭취가 감소하는 등 식품 및 영양소 섭취 양상은 변화가 있었다. 코로나19 유행 전, 후를 비교 시, 외식 감소, 가정식 섭취 증가 등 식생활은 변화가 있었으나 식품 및 영양소 섭취는 개선 또는 악화되지 않은 채 10년간의 추이가 지속되고 있었다. 코로나19 유행으로 인해 식품 및 영양소의 섭취가 변화가 없었다는 점은 고무적이나, 코로나19 유행이 지속되는 상황을 고려하여 식생활 변화가 식품 및 영양소 섭취 수준에 미치는 영향 여부 및 관련요인에 관한 모니터링이 필요하다.

**REFERENCES**

1. Antwi J, Appiah B, Oluwakuse B, Abu BA. The nutrition-COVID-19 interplay: a review. Curr Nutr Rep 2021;10:364-374.

2. Rubio-Tomás T, Skouroliakou M, Ntountaniotis D. Lockdown due to COVID-19 and its consequences on diet, physical activity, lifestyle, and other aspects of daily life worldwide: a narrative re­view. Int J Environ Res Public Health 2022;19:6832.

3. Mignogna C, Costanzo S, Ghulam A, Cerletti C, Donati MB, de Gaetano G, et al. Impact of nationwide lockdowns resulting from the first wave of the COVID-19 pandemic on food intake, eating behaviours and diet quality: a systematic review. Adv Nutr 2021;13:388–423.

4. Merino J, Joshi AD, Nguyen LH, Leeming ER, Mazidi M, Drew DA, et al. Diet quality and risk and severity of COVID-19: a pro­spective cohort study. Gut 2021;70:2096-2104.

5. Yue Y, Ma W, Accorsi EK, Ding M, Hu F, Willett WC, et al. Long-term diet and risk of severe acute respiratory syndrome corona­virus 2 (SARS-CoV-2) infection and coronavirus disease 2019 (COVID-19) severity. Am J Clin Nutr 2022;116:1672-1681.

6. Stefan N, Birkenfeld AL, Schulze MB. Global pandemics inter­connected - obesity, impaired metabolic health and COVID-19. Nat Rev Endocrinol 2021;17:135-149.

7. Detopoulou P, Tsouma C, Papamikos V. COVID-19 and nutrition: summary of official recommendations. Top Clin Nutr 2022;37:187-202.

8. Deschasaux-Tanguy M, Druesne-Pecollo N, Esseddik Y, de Edele­nyi FS, Allès B, Andreeva VA, et al. Diet and physical activity dur­ing the coronavirus disease 2019 (COVID-19) lockdown (March-May 2020): results from the French NutriNet-Santé cohort study. Am J Clin Nutr 2021;113:924-938.

9. Ammar A, Brach M, Trabelsi K, Chtourou H, Boukhris O, Mas­moudi L, et al. Effects of COVID-19 home confinement on eating behaviour and physical activity: results of the ECLB-COVID19 international online survey. Nutrients 2020;12:1583.

10. Bhutani S, vanDellen MR, Cooper JA. Longitudinal weight gain and related risk behaviors during the COVID-19 pandemic in adults in the US. Nutrients 2021;13:671.

11. Poskute AS, Nzesi A, Geliebter A. Changes in food intake during the COVID-19 pandemic in New York City. Appetite 2021;163:105191.

12. Cheikh Ismail L, Osaili TM, Mohamad MN, Al Marzouqi A, Jar­rar AH, Zampelas A, et al. Assessment of eating habits and life­style during the coronavirus 2019 pandemic in the Middle East and North Africa region: a cross-sectional study. Br J Nutr 2021;126:757-766.

13. Salman A, Sigodo KO, Al-Ghadban F, Al-Lahou B, Alnashmi M, Hermassi S, et al. Effects of COVID-19 lockdown on physical ac­tivity and dietary behaviors in Kuwait: a cross-sectional study. Nutrients 2021;13:2252.

14. Bogataj Jontez N, Novak K, Kenig S, Petelin A, Jenko Pražnikar Z, Mohorko N. The impact of COVID-19-related lockdown on diet and serum markers in healthy adults. Nutrients 2021;13:1082.

15. Cosgrove K, Wharton C. Predictors of COVID-19-related per­ceived improvements in dietary health: results from a US cross-sectional study. Nutrients 2021;13:2097.

16. Hori N, Shiraishi M, Harada R, Kurashima Y. Association of life­style changes due to the COVID-19 pandemic with nutrient in­take and physical activity levels during pregnancy in Japan. Nu­trients 2021;13:3799.

17. Shimpo M, Akamatsu R, Kojima Y, Yokoyama T, Okuhara T, Chi­ba T. Factors associated with dietary change since the outbreak of COVID-19 in Japan. Nutrients 2021;13:2039.

18. Choi S, Kim Y, Yang J, Oh K. Health behaviors among Korean adolescents before and during the COVID-19 pandemic; 2022 [cited 2022 Sep 1]. Available from: https://www.kdca.go.kr/yhs/yhshmpg/ntcn/bbsCntntsdetail.do?bbsCode=B00003&bbscttNo=1641 (Korean).

19. Lee GB, Kim Y, Park S, Kim HC, Oh K. Obesity, hypertension, diabetes mellitus, and hypercholesterolemia in Korean adults be­fore and during the COVID-19 pandemic: a special report of the 2020 Korea National Health and Nutrition Examination Survey. Epidemiol Health 2022;44:e2022041.

20. Choi S, Bahk J, Park S, Oh K, Jung-Choi K. Smoking, drinking, and physical activity among Korean adults before and during the COVID-19 pandemic: a special report of the 2020 Korea Nation­al Health and Nutrition Examination Survey. Epidemiol Health 2022;44:e2022043.

21. Korea Disease Control and Prevention Agency. 2020 The Korea Health Community Survey: Physical activity decreased and personal hygiene improved by region.; 2021 Apr 1 [cited 2022 Sep 10]. Available from: https://www.korea.kr/news/pressReleaseView.do?newsId=156444291 (Korean).

22. Korea Disease Control and Prevention Agency (KDCA). Sample design for 2021 National Health Behavior Survey 2021. Cheongju: KDCA; 2021 (Korean).

23. Kweon S, Park JY, Park M, Kim Y, Yeon SY, Yoon L, et al. Trends in food and nutrient intake over 20 years: findings from the 1998-2018 Korea National Health and Nutrition Examination Survey. Epidemiol Health 2021;43:e2021027.

24. Popkin BM, Ng SW. The nutrition transition to a stage of high obesity and noncommunicable disease prevalence dominated by ultra-processed foods is not inevitable. Obes Rev 2022;23:e13366.

25. Saito A, Imai S, Htun NC, Okada E, Yoshita K, Yoshiike N, et al. The trends in total energy, macronutrients and sodium intake among Japanese: findings from the 1995-2016 National Health and Nutrition Survey. Br J Nutr 2018;120:424-434.

26. National Institute of Health and Nutrition of Japan. National Health and Nutrition Survey [cited 2022 Sep 10]. Available from: https://www.nibiohn.go.jp/eiken/kenkounippon21/en/eiyouchousa/kekka_eiyou_chousa.html.

27. Dokova KG, Pancheva RZ, Usheva NV, Haralanova GA, Nikolova SP, Kostadinova TI, et al. Nutrition transition in Europe: East-West dimensions in the last 30 years-a narrative review. Front Nutr 2022;9:919112.

28. Shan Z, Rehm CD, Rogers G, Ruan M, Wang DD, Hu FB, et al. Trends in dietary carbohydrate, protein, and fat intake and diet quality among US adults, 1999-2016. JAMA 2019;322:1178-1187.

29. Lee G, Kim S, Hur S, Shin S, Park I. The consumer behavior sur­vey for food 2020. Naju: Korea Rural Economic Institute; 2020 (Korean).

30. Lee G, Kim S, Shin S, Shim H, Park I. The Consumer Behavior Survey for Food 2021. Naju: Korea Rural Economic Institute; 2021 (Korean).

31. Zhang J, Zhao A, Ke Y, Huo S, Ma Y, Zhang Y, et al. Dietary be­haviors in the post-lockdown period and its effects on dietary di­versity: the second stage of a nutrition survey in a longitudinal Chinese study in the COVID-19 era. Nutrients 2020;12:3269.

32. Cicero AF, Fogacci F, Giovannini M, Mezzadri M, Grandi E, Borghi C, The Brisighella Heart Study Group. COVID-19-related quar­antine effect on dietary habits in a northern Italian rural popula­tion: data from the Brisighella Heart Study. Nutrients 2021;13:309.

33. Yang GY, Lin XL, Fang AP, Zhu HL. Eating Habits and lifestyles during the initial stage of the COVID-19 lockdown in China: a cross-sectional study. Nutrients 2021;13:970.

34. Public Health England. National diet and nutrition survey: diet, nutrition and physical activity in 2020. A follow up study during COVID-19; 2021 [cited 2022 Sep 10]. Available from: https://as­sets.publishing.service.gov.uk/government/uploads/system/up­loads/attachment_data/file/1019663/Follow_up_stud_2020_main_report.pdf.

35. Acedo C, Roncero-Martín R, Sánchez-Fernández A, Mendoza-Holgado C, Pedrera-Canal M, López-Espuela F, et al. Body com­position and nutrients dietary intake changes during COVID-19 lockdown in Spanish healthy postmenopausal women. Eur J In­vestig Health Psychol Educ 2022;12:631-638.

36. Perrar I, Alexy U, Jankovic N. Changes in total energy, nutrients and food group intake among children and adolescents during the COVID-19 pandemic-results of the DONALD study. Nutri­ents 2022;14:297.

Table 1. General characteristics of the participants in Korean men and women aged 19 or older in the 2011-2020 Korea National Health and Nutrition Examination Survey (KNHANES)

|  | 2011 | | 2012 | | 2013 | | 2014 | | 2015 | | 2016 | | 2017 | | 2018 | | 2019 | | 2020 | | |
| --- | --- | --- | --- | --- | --- | --- | --- | --- | --- | --- | --- | --- | --- | --- | --- | --- | --- | --- | --- | --- | --- |
|  | n | % | n | % | n | % | n | % | n | % | n | % | n | % | n | % | n | % | n | % |  |
| Total | 5,894 |  | 5,570 |  | 5,450 |  | 5,323 |  | 5,308 |  | 5,439 |  | 5,723 |  | 5,708 |  | 5,775 |  | 4,805 |  |  |
| Sex |  |  |  |  |  |  |  |  |  |  |  |  |  |  |  |  |  |  |  |  |  |
| Men | 2,434 | 49.4 | 2,231 | 49.3 | 2,269 | 49.5 | 2,199 | 49.6 | 2,249 | 49.4 | 2,245 | 49.7 | 2,484 | 49.7 | 2,443 | 49.8 | 2,498 | 49.8 | 2,085 | 49.6 |  |
| Women | 3,460 | 50.6 | 3,339 | 50.7 | 3,181 | 50.5 | 3,124 | 50.4 | 3,059 | 50.6 | 3,194 | 50.3 | 3,239 | 50.3 | 3,265 | 50.2 | 3,277 | 50.2 | 2,720 | 50.4 |  |
| Age (yr) |  |  |  |  |  |  |  |  |  |  |  |  |  |  |  |  |  |  |  |  |  |
| 19-29 | 643 | 19.0 | 582 | 18.6 | 673 | 18.3 | 567 | 18.3 | 661 | 18.4 | 623 | 17.7 | 653 | 17.5 | 682 | 17.6 | 669 | 17.3 | 624 | 17.3 |  |
| 30-39 | 1,054 | 20.7 | 937 | 20.2 | 964 | 19.7 | 908 | 19.2 | 718 | 18.6 | 966 | 18.3 | 845 | 17.9 | 862 | 17.5 | 855 | 17.1 | 633 | 16.5 |  |
| 40-49 | 1,027 | 21.8 | 901 | 21.6 | 1,064 | 21.3 | 899 | 21.0 | 904 | 20.6 | 1,014 | 20.8 | 1,028 | 20.4 | 1,052 | 19.9 | 1,036 | 19.4 | 808 | 19.0 |  |
| 50-59 | 1,145 | 18.1 | 1,034 | 18.7 | 1,002 | 19.3 | 995 | 19.6 | 1,081 | 19.7 | 939 | 19.9 | 1,117 | 19.9 | 1,055 | 19.8 | 1,061 | 20.0 | 861 | 19.7 |  |
| 60-69 | 995 | 10.6 | 1,013 | 10.7 | 874 | 10.9 | 926 | 11.3 | 960 | 11.8 | 920 | 12.4 | 1,037 | 12.9 | 998 | 13.5 | 1,046 | 14.1 | 892 | 14.9 |  |
| ≥70 | 1,030 | 9.8 | 1,103 | 10.2 | 873 | 10.5 | 1,028 | 10.7 | 984 | 10.9 | 977 | 10.9 | 1,043 | 11.3 | 1,059 | 11.7 | 1,108 | 12.2 | 987 | 12.6 |  |
| Household income^1^ |  |  |  |  |  |  |  |  |  |  |  |  |  |  |  |  |  |  |  |  |  |
| Low | 1,157 | 22.6 | 1,087 | 22.2 | 1,068 | 19.9 | 1,074 | 21.1 | 1,052 | 20.2 | 1,091 | 20.8 | 1,150 | 19.6 | 1,146 | 20.4 | 1,151 | 19.3 | 963 | 19.4 |  |
| Low-middle | 1,182 | 20.6 | 1,119 | 21.3 | 1,088 | 19.9 | 1,041 | 19.8 | 1,071 | 20.3 | 1,064 | 18.9 | 1,155 | 20.4 | 1,141 | 20.8 | 1,129 | 19.6 | 967 | 19.3 |  |
| Middle | 1,167 | 19.9 | 1,078 | 20.3 | 1,108 | 20.7 | 1,054 | 19.3 | 1,038 | 19.6 | 1,077 | 20.0 | 1,138 | 19.1 | 1,132 | 20.1 | 1,176 | 20.4 | 964 | 19.9 |  |
| Middle-high | 1,169 | 19.3 | 1,102 | 18.2 | 1,085 | 20.2 | 1,055 | 19.6 | 1,053 | 20.0 | 1,084 | 19.6 | 1,163 | 21.0 | 1,140 | 19.8 | 1,150 | 20.3 | 927 | 20.0 |  |
| High | 1,153 | 17.7 | 1,086 | 18.0 | 1,060 | 19.3 | 1,076 | 20.2 | 1,060 | 20.0 | 1,102 | 20.7 | 1,103 | 19.8 | 1,137 | 18.9 | 1,145 | 20.3 | 967 | 21.4 |  |

Values are presented as number and weighted %

^1^Calculated as monthly household income divided by square root of the number of persons in the household, categorized into quintiles according to age and gender.

Table 2. Trends in dietary habits in Korean men and women aged 19 or older in the 2011-2020 Korea National Health and Nutrition Examination Survey (KNHANES)

| Variables | Category | 2011 | 2012 | 2013 | 2014 | 2015 | 2016 | 2017 | 2018 | 2019 | 2020 | Trend^1^  (2011-2020) | | Trend^1^  (2011-2019) | | Difference^1^  (2019 to 2020) | | |
| --- | --- | --- | --- | --- | --- | --- | --- | --- | --- | --- | --- | --- | --- | --- | --- | --- | --- | --- |
|  |  |  |  |  |  |  |  |  |  |  |  | β estimate | p value | β estimate | p value | difference | | p value |
| Skipping breakfast(%)^2^ | Total | 22.5 | 24.6 | 25.2 | 25.6 | 28.0 | 29.6 | 29.8 | 30.9 | 34.1 | 36.7 | 0.013 | <.0001 | 0.012 | <.0001 | 2.6 | 0.080 | |
|  | Men | 22.9 | 23.5 | 27.2 | 27.5 | 29.5 | 32.4 | 33.0 | 30.6 | 35.4 | 38.8 | 0.015 | <.0001 | 0.014 | <.0001 | 3.4 | 0.081 | |
|  | Women | 22.0 | 25.7 | 22.9 | 23.5 | 26.2 | 26.4 | 26.4 | 31.2 | 32.6 | 34.3 | 0.012 | <.0001 | 0.010 | <.0001 | 1.7 | 0.343 | |
| Eating out more than once a day(%)^3^ | Total | 29.4 | 25.6 | 31.9 | 30.6 | 31.3 | 32.3 | 30.1 | 33.5 | 31.0 | 26.4 | 0.001 | 0.313 | 0.004 | 0.0001 | -4.6 | 0.001 | |
|  | Men | 41.6 | 36.5 | 45.2 | 43.3 | 43.1 | 45.8 | 41.2 | 44.8 | 41.0 | 35.8 | -0.002 | 0.119 | 0.002 | 0.325 | -5.2 | 0.012 | |
|  | Women | 16.8 | 14.4 | 18.1 | 17.3 | 19.2 | 18.3 | 18.4 | 21.8 | 20.5 | 16.7 | 0.004 | <.0001 | 0.006 | <.0001 | -3.8 | 0.014 | |
| Dinner with family members(%)^4^ | Total | 62.7 | 63.0 | 61.3 | 61.6 | 60.6 | 60.5 | 61.3 | 61.3 | 64.5 | 65.1 | 0.001 | 0.376 | -0.001 | 0.673 | 0.6 | 0.735 | |
|  | Men | 58.9 | 62.6 | 57.7 | 57.0 | 58.1 | 56.2 | 57.6 | 58.3 | 60.4 | 63.3 | 0.001 | 0.507 | -0.002 | 0.402 | 2.9 | 0.198 | |
|  | Women | 67.2 | 63.9 | 65.6 | 67.0 | 63.6 | 65.6 | 65.7 | 65.0 | 69.4 | 67.4 | 0.001 | 0.451 | 0.000 | 0.761 | -2.0 | 0.271 | |
| Household with food security(%)^5^ | Total | 95.2 | 92.2 | 93.5 | 93.8 | 93.5 | 95.8 | 96.3 | 96.9 | 96.5 | 96.3 | 0.002 | 0.002 | 0.002 | 0.003 | -0.2 | 0.658 | |

Values are presented as weighted percentage (%); Age-standardized prevalence was calculated using the 2005 Census Korean.

^1^ Values for trend and difference are adjusted for age and household income.

^2^ the proportion of population skipping breakfast one day before the survey

^3^ the proportion of population eating out more than once a day

^4^ the proportion of population having dinner with family members

^5^ the proportion of households that responded 'all members of our family were able to eat enough amount and various kinds of foods as much as we need' or 'all members of our family were able to eat enough amount, but we were not able to eat various kinds of foods'

Table 3. Trends in the major food groups among Korean men and women aged 19 or older in the 2011-2020 Korea National Health and Nutrition Examination Survey (KNHANES)

| Variables | Category | 2011 | 2012 | 2013 | 2014 | 2015 | 2016 | 2017 | 2018 | 2019 | 2020 | Trend^1^  (2011-2020) | | Trend^1^  (2011-2019) | | Difference^1^  (2019 to 2020) | |
| --- | --- | --- | --- | --- | --- | --- | --- | --- | --- | --- | --- | --- | --- | --- | --- | --- | --- |
|  |  |  |  |  |  |  |  |  |  |  |  | β estimate | p value | β estimate | p value | difference | p value |
| Grains  (g/day) | Total | 312.3 | 304.4 | 303.3 | 296.0 | 304.4 | 295.9 | 290.1 | 293.4 | 274.4 | 271.0 | -4.221 | <.0001 | -3.768 | <.0001 | -3.4 | 0.468 |
|  | Men | 350.8 | 342.3 | 337.8 | 332.6 | 346.9 | 335.0 | 325.2 | 335.6 | 314.2 | 310.0 | -3.857 | <.0001 | -3.300 | <.0001 | -4.3 | 0.531 |
|  | Women | 273.5 | 266.3 | 268.1 | 258.7 | 261.2 | 255.9 | 254.4 | 249.7 | 233.4 | 230.9 | -4.736 | <.0001 | -4.412 | <.0001 | -2.5 | 0.636 |
| Vegetables  (g/day) | Total | 328.2 | 322.9 | 320.0 | 328.4 | 317.8 | 293.8 | 299.8 | 275.8 | 283.7 | 276.2 | -6.086 | <.0001 | -6.167 | <.0001 | -7.5 | 0.187 |
|  | Men | 376.7 | 361.5 | 361.7 | 365.3 | 361.5 | 335.5 | 338.8 | 315.9 | 323.8 | 312.8 | -7.017 | <.0001 | -6.945 | <.0001 | -10.9 | 0.172 |
|  | Women | 279.7 | 284.8 | 278.5 | 291.4 | 273.4 | 252.2 | 260.1 | 235.2 | 242.5 | 239.1 | -5.371 | <.0001 | -5.622 | <.0001 | -3.4 | 0.542 |
| Fruits  (g/day) | Total | 178.0 | 175.5 | 175.0 | 188.2 | 200.2 | 176.0 | 156.4 | 134.5 | 140.6 | 124.9 | -5.928 | <.0001 | -4.787 | <.0001 | -15.8 | 0.054 |
|  | Men | 163.8 | 161.0 | 156.7 | 161.4 | 181.4 | 163.6 | 139.1 | 120.5 | 124.4 | 111.4 | -5.841 | <.0001 | -4.872 | <.0001 | -13.1 | 0.116 |
|  | Women | 192.7 | 190.2 | 194.4 | 215.9 | 219.6 | 189.2 | 174.1 | 149.1 | 157.3 | 139.0 | -5.951 | <.0001 | -4.630 | <.0001 | -18.3 | 0.094 |
| Meat  (g/day) | Total | 109.6 | 115.0 | 104.9 | 105.6 | 109.8 | 114.4 | 117.7 | 118.9 | 125.2 | 128.4 | 1.962 | <.0001 | 1.616 | <.0001 | 3.3 | 0.489 |
|  | Men | 136.3 | 146.4 | 130.6 | 128.2 | 137.2 | 145.9 | 149.4 | 149.3 | 159.2 | 165.2 | 2.924 | <.0001 | 2.376 | 0.0003 | 6.0 | 0.420 |
|  | Women | 82.5 | 82.6 | 78.3 | 82.3 | 81.3 | 81.0 | 84.1 | 86.6 | 88.9 | 89.6 | 0.921 | 0.002 | 0.743 | 0.032 | 0.7 | 0.861 |
| Non-alcoholic beverages(g/day) | Total | 129.6 | 141.0 | 186.2 | 192.2 | 210.9 | 231.8 | 229.0 | 230.6 | 247.0 | 254.3 | 12.394 | <.0001 | 13.247 | <.0001 | 7.3 | 0.517 |
|  | Men | 141.3 | 152.7 | 206.7 | 214.8 | 240.8 | 263.4 | 252.2 | 249.0 | 272.6 | 277.6 | 13.768 | <.0001 | 14.751 | <.0001 | 5.0 | 0.771 |
|  | Women | 118.0 | 129.2 | 165.0 | 168.3 | 179.2 | 197.8 | 204.3 | 210.2 | 219.5 | 230.0 | 10.988 | <.0001 | 11.678 | <.0001 | 10.5 | 0.345 |

Values are presented as weighted mean intake (unit: g/d); Age-standardized mean was calculated using the 2005 Census Korean.

^1^ Values for trend and difference are adjusted for age and household income.

Table 4. Trends in energy intake among Korean men and women aged 19 or older in the 2011-2020 Korea National Health and Nutrition Examination Survey (KNHANES)

| Variables | | Category | 2011 | 2012 | 2013 | 2014 | 2015 | 2016 | 2017 | 2018 | 2019 | 2020 | Trend^1^  (2011-2020) | | Trend^1^  (2011-2019) | | Difference^1^  (2019 to 2020) | |
| --- | --- | --- | --- | --- | --- | --- | --- | --- | --- | --- | --- | --- | --- | --- | --- | --- | --- | --- |
|  |  |  |  |  |  |  |  |  |  |  |  |  | βestimate | p value | βestimate | p value | difference | p value |
| Energy  (kcal) | | Total | 2,090.9 | 2,057.1 | 2,135.7 | 2,121.7 | 2,174.8 | 2,112.4 | 2,071.8 | 2,042.6 | 1993.0 | 1,953.4 | -14.505 | <.0001 | -9.862 | <.0001 | -39.6 | 0.159 |
|  |  | Men | 2,464.3 | 2,412.5 | 2,477.5 | 2,454.6 | 2,538.7 | 2,491.1 | 2408.0 | 2,398.2 | 2,338.1 | 2,281.4 | -17.512 | <.0001 | -11.702 | 0.001 | -56.7 | 0.175 |
|  |  | Women | 1,715.4 | 1,698.5 | 1,787.2 | 1,781.7 | 1,803.7 | 1,722.1 | 1,724.7 | 1,673.6 | 1,634.4 | 1,614.7 | -12.987 | <.0001 | -9.771 | <.0001 | -19.7 | 0.464 |
| Energy-  source  nutrient^2^ | Fat(%) | Total | 19.2 | 19.8 | 20.4 | 21.0 | 21.1 | 21.2 | 21.5 | 22.0 | 23.1 | 24.1 | 0.477 | <.0001 | 0.417 | <.0001 | 1.0 | 0.002 |
|  |  | Men | 19.9 | 20.5 | 20.9 | 21.5 | 21.5 | 21.8 | 21.8 | 22.3 | 23.4 | 24.3 | 0.417 | <.0001 | 0.354 | <.0001 | 0.9 | 0.026 |
|  |  | Women | 18.6 | 19.1 | 19.9 | 20.5 | 20.7 | 20.6 | 21.2 | 21.7 | 22.7 | 23.8 | 0.533 | <.0001 | 0.477 | <.0001 | 1.0 | 0.003 |
|  | Protein(%) | Total | 14.9 | 14.9 | 14.6 | 14.7 | 14.7 | 15.3 | 15.4 | 15.5 | 15.9 | 15.9 | 0.133 | <.0001 | 0.126 | <.0001 | 0.0 | 0.939 |
|  |  | Men | 15.4 | 15.4 | 15.2 | 15.1 | 15.2 | 15.9 | 15.9 | 16.0 | 16.3 | 16.5 | 0.131 | <.0001 | 0.120 | <.0001 | 0.2 | 0.499 |
|  |  | Women | 14.3 | 14.5 | 14.0 | 14.2 | 14.2 | 14.7 | 14.8 | 15.0 | 15.4 | 15.2 | 0.132 | <.0001 | 0.128 | <.0001 | -0.1 | 0.449 |
|  | Carbohydrates(%) | Total | 65.9 | 65.3 | 65.0 | 64.3 | 64.2 | 63.5 | 63.1 | 62.5 | 61.1 | 60.1 | -0.609 | <.0001 | -0.543 | <.0001 | -1.0 | 0.008 |
|  |  | Men | 64.7 | 64.2 | 63.8 | 63.3 | 63.4 | 62.3 | 62.2 | 61.7 | 60.3 | 59.2 | -0.548 | <.0001 | -0.474 | <.0001 | -1.1 | 0.021 |
|  |  | Women | 67.1 | 66.4 | 66.0 | 65.2 | 65.1 | 64.7 | 64.0 | 63.3 | 61.9 | 61.0 | -0.665 | <.0001 | -0.605 | <.0001 | -0.9 | 0.047 |
| Meal  type | Home-cooked meal  (%) | Total | - | - | - | - | - | 39.7 | 39.6 | 39.2 | 37.5 | 39.9 | -0.207 | 0.308 | -0.786 | 0.004 | 2.5 | 0.011 |
|  |  | Men | - | - | - | - | - | 35.9 | 35.8 | 36.2 | 34.6 | 37.0 | 0.065 | 0.807 | -0.357 | 0.314 | 2.5 | 0.057 |
|  |  | Women | - | - | - | - | - | 43.7 | 43.6 | 42.3 | 40.4 | 42.9 | -0.456 | 0.052 | -1.171 | 0.0002 | 2.6 | 0.019 |
|  | Restaurant meal  (%) | Total | - | - | - | - | - | 31.8 | 33.0 | 32.5 | 33.5 | 31.9 | 0.109 | 0.587 | 0.478 | 0.072 | -1.5 | 0.180 |
|  |  | Men | - | - | - | - | - | 36.4 | 37.2 | 35.7 | 36.7 | 35.1 | -0.220 | 0.436 | -0.040 | 0.917 | -1.5 | 0.282 |
|  |  | Women | - | - | - | - | - | 27.0 | 28.7 | 29.2 | 30.3 | 28.7 | 0.420 | 0.056 | 0.965 | 0.001 | -1.4 | 0.243 |
|  | Food services meal at shools or workplace (%) | Total | - | - | - | - | - | 5.2 | 5.2 | 5.6 | 5.8 | 3.8 | -0.222 | 0.021 | 0.212 | 0.132 | -2.0 | <.0001 |
|  |  | Men | - | - | - | - | - | 6.7 | 6.3 | 6.7 | 7.2 | 4.5 | -0.350 | 0.019 | 0.183 | 0.417 | -2.8 | 0.0001 |
|  |  | Women | - | - | - | - | - | 3.5 | 4.0 | 4.5 | 4.2 | 3.1 | -0.105 | 0.222 | 0.217 | 0.069 | -1.1 | 0.013 |
|  | Convenience food(%) | Total | - | - | - | - | - | 7.3 | 7.4 | 8.3 | 8.5 | 9.7 | 0.591 | <.0001 | 0.434 | 0.0003 | 1.2 | 0.014 |
|  |  | Men | - | - | - | - | - | 7.8 | 7.9 | 8.8 | 8.9 | 10.3 | 0.617 | <.0001 | 0.474 | 0.006 | 1.4 | 0.052 |
|  |  | Women | - | - | - | - | - | 6.9 | 7.0 | 7.6 | 8.1 | 9.1 | 0.562 | <.0001 | 0.393 | 0.005 | 1.1 | 0.069 |

Values are presented as weighted mean intake or weighted percentage (%); Age-standardized mean was calculated using the 2005 Census Korean.

^1^Values for trend and difference are adjusted for age and household income. For meal type, trends were calculated using 2016-2020 and 2016-2019 data, respectively.

^2^The percentage of energy from fat means the percentage of energy from fat (g of fat×9 kcal/g) compared to the sum of energy from fat, carbohydrates, and protein; The respective percentages of energy from the other components were calculated using a similar equation.

Table 5. Trends in nutrient intakes among Korean men and women aged 19 or older in the 2011-2020 Korea National Health and Nutrition Examination Survey

| Variables | Category | 2011 | 2012 | 2013 | 2014 | 2015 | 2016 | 2017 | 2018 | 2019 | 2020 | Trend^1^  (2011-2020) | | | Trend^1^  (2011-2019) | | | Difference^1^  (2019 to 2020) | |
| --- | --- | --- | --- | --- | --- | --- | --- | --- | --- | --- | --- | --- | --- | --- | --- | --- | --- | --- | --- |
|  |  |  |  |  |  |  |  |  |  |  |  | β stimate | p value | | βestimate | p value | | difference | p value |
| Calcium  (mg/d) | Total | 518.0 | 508.3 | 500.3 | 494.6 | 510.1 | 526.5 | 524.4 | 517.3 | 503.6 | 493.9 | 0.145 | | 0.830 | 1.349 | | 0.088 | -9.7 | 0.246 |
|  | Men | 581.1 | 546.2 | 552.6 | 544.7 | 568.1 | 587.6 | 569.6 | 580.2 | 550.3 | 536.8 | -0.642 | | 0.502 | 1.317 | | 0.240 | -13.5 | 0.243 |
|  | Women | 454.6 | 470.7 | 448.4 | 443.8 | 451.3 | 464.7 | 478.7 | 453.0 | 456.3 | 450.0 | 0.677 | | 0.368 | 1.100 | | 0.220 | -6.3 | 0.522 |
| Sodium  (mg/d) | Total | 5,211.0 | 4,942.1 | 4,176.3 | 4,033.2 | 4,188.7 | 3,585.5 | 3,586.5 | 3,488.4 | 3,512.3 | 3,412.6 | -184.720 | | <.0001 | -207.719 | | <.0001 | -99.7 | 0.103 |
|  | Men | 6,177.9 | 5,726.9 | 4,891.0 | 4,698.1 | 4,984.6 | 4,244.5 | 4,171.1 | 4,133.5 | 4,164.6 | 4,037.5 | -216.195 | | <.0001 | -242.554 | | <.0001 | -127.2 | 0.134 |
|  | Women | 4,241.6 | 4,154.3 | 3,448.3 | 3,354.2 | 3,379.9 | 2,910.4 | 2,989.8 | 2,820.8 | 2,836.0 | 2,769.3 | -156.859 | | <.0001 | -177.062 | | <.0001 | -66.7 | 0.260 |
| Iron  (mg/d) | Total | 15.4 | 15.3 | 18.2 | 17.9 | 17.8 | 12.5 | 12.2 | 12.1 | 11.8 | 11.2 | -0.718 | | <.0001 | -0.712 | | <.0001 | -0.6 | 0.003 |
|  | Men | 17.5 | 16.9 | 21.0 | 20.4 | 20.2 | 14.1 | 13.7 | 13.9 | 13.6 | 12.9 | -0.801 | | <.0001 | -0.793 | | <.0001 | -0.7 | 0.026 |
|  | Women | 13.2 | 13.6 | 15.3 | 15.4 | 15.3 | 10.7 | 10.7 | 10.2 | 10.0 | 9.5 | -0.644 | | <.0001 | -0.642 | | <.0001 | -0.6 | 0.011 |
| Vitamin A  (μgRAE/d) | Total | - | - | - | - | - | 403.4 | 381.7 | 383.6 | 379.9 | 396.0 | -1.035 | | 0.745 | -4.865 | | 0.237 | 16.1 | 0.207 |
|  | Men | - | - | - | - | - | 444.6 | 406.3 | 420.1 | 410.6 | 418.4 | -5.728 | | 0.157 | -8.334 | | 0.116 | 7.8 | 0.643 |
|  | Women | - | - | - | - | - | 362.1 | 357.3 | 346.5 | 348.4 | 373.0 | 3.461 | | 0.360 | -1.809 | | 0.724 | 24.6 | 0.065 |
| Riboflavin (μg/d) | Total | 1,297.7 | 1,312.3 | 1,415.0 | 1,443.7 | 1,466.4 | 1,657.3 | 1,645.5 | 1,667.9 | 1,694.4 | 1,756.4 | 52.222 | | <.0001 | 53.704 | | <.0001 | 62.0 | 0.040 |
|  | Men | 1,493.7 | 1,486.1 | 1,600.3 | 1,643.7 | 1,655.1 | 1,907.2 | 1,860.8 | 1,909.4 | 1,916.2 | 2,009.1 | 59.071 | | <.0001 | 60.475 | | <.0001 | 92.9 | 0.031 |
|  | Women | 1,105.0 | 1,136.8 | 1,227.5 | 1,237.5 | 1,274.7 | 1,398.4 | 1,424.1 | 1,416.9 | 1,463.5 | 1,494.1 | 44.641 | | <.0001 | 46.019 | | <.0001 | 30.5 | 0.300 |
| Vitamin C  (mg/d) | Total | 109.2 | 110.0 | 95.2 | 101.8 | 100.7 | 62.8 | 63.5 | 61.3 | 67.0 | 64.2 | -6.408 | | <.0001 | -7.193 | | <.0001 | -2.8 | 0.302 |
|  | Men | 115.2 | 114.0 | 92.0 | 96.5 | 97.8 | 63.0 | 64.2 | 67.6 | 67.7 | 68.0 | -6.310 | | <.0001 | -7.248 | | <.0001 | 0.3 | 0.936 |
|  | Women | 103.3 | 106.2 | 98.6 | 107.3 | 103.7 | 62.8 | 62.7 | 54.7 | 66.1 | 60.2 | -6.510 | | <.0001 | -7.143 | | <.0001 | -5.9 | 0.040 |

Values are presented as weighted mean intake; Age-standardized mean was calculated using the 2005 Census Korean.

^1^Values for trend and difference are adjusted for age and household income. For vitamin A, trends were calculated using 2016-2020 and 2016-2019 data, respectively.
